# Multiple aneurysms and gastrointestinal involvement in Behcet's disease: A case report: Erratum

**DOI:** 10.1097/MD.0000000000009475

**Published:** 2017-12-22

In the article, “Multiple aneurysms and gastrointestinal involvement in Behcet's disease: A case report”,^[1]^ which appeared in Volume 96, Issue 49 of *Medicine*, the authors would like to correct the reported degrees for each author. They should have been listed as: Mengdi Wang, MM, Weiwei Sun,MD, Zhenjie Chen, MD, Xiaona Wang, BM, Jie Lv, MD, Quanming Tan, MM, Yaoxian Wang, MD, Jingwei Zhou, MD.
